# Effect of esketamine on postpartum depression after labor analgesia and potential mechanisms: a randomized, double-blinded controlled trial

**DOI:** 10.1186/s12871-023-02377-6

**Published:** 2024-01-02

**Authors:** Wei Wang, Bin Ling, Haibo Zhao, Jing He, Hua Xu, Jie Lv, Qi Wang

**Affiliations:** 1 Department of Anesthesiology, The Affiliated Jiangning Hospital of Nanjing Medical University, No. 169 Hushan Road, Nanjing, 211100 China; 2 Department of Gynaecology and obstetrics, The Affiliated Jiangning Hospital of Nanjing Medical University, Nangjing, 211100 China

**Keywords:** Postpartum depression, Esketamine, Labor analgesia, Epidural anaesthesia, Leptin

## Abstract

**Background:**

To evaluate the effect of esketamine combined with ropivacaine hydrochloride on the occurrence of postpartum depression (PPD) after labor analgesia under epidural analgesia pump and explore the possible mechanisms.

**Methods:**

A total of 120 women aged 24 to 36 years old who underwent labor analgesia by epidural analgesia pump, with American Society of Anesthesiologists (ASA) physical status II were enrolled. According to the formula of epidural analgesia pump, all participants were randomly divided into two groups: esketamine group (Group E) and control group (Group C). Epidural anaesthesia were operated in all women between L_2_ and L_3_ after cervical dilation up to 2 ~ 3 cm. After successful puncture, the epidural catheter was placed 3.5 cm toward the head and 1% lidocaine was injected for 3 ml. The epidural analgesia pump was connected. Esketamine (0.2 mg/kg) combined with 0.75% ropivacaine hydrochloride (20 ml) were diluted by normal saline up to 100 ml in Group E, when only the equal dose of ropivacaine hydrochloride was used in Group C. The visual analogue scale (VAS) before analgesia (T_1_), 5 (T_2_), 10 (T_3_) and 20 (T_4_) minutes after analgesia were measured. The duration of the first and second stage of labor, the Apgar score of fetus at delivery, postpartum hemorrhage, consumption of esketamine and ropivacaine were recorded. The incidence of PPD was recorded at 1 week and 6 weeks after delivering. The occurrence of side effects such as nausea and vomiting, dizziness, and nightmares were also recorded for 48 h after delivering. The levels of leptin, norepinephrine(NE), and epinephrine(E) in the peripheral venous blood were measured before labor analgesia and at 24 h, 1 week, and 6 weeks after delivering.

**Results:**

Compared with Group C, the VAS score at T_2_, T_3_ and T_4_ were significantly lower in Group E (*P* < 0.01). Compared with Group C, the incidence of PPD was significantly lower at 1 week and 6 weeks after delivering in Group E (*P* < 0.01). Compared with Group C, the levels of leptin were significantly higher at 24 h and 1 week after delivering in Group E (*P* < 0.01), while NE and E (*P* < 0.01) were lower at the same time (*P* < 0.01). There were no significant difference of the duration of the first and second stage of labor, the Apgar score of fetus at delivery, postpartum hemorrhage, consumption of ropivacaine and the side effects for 48 h after delivering between the two groups.

**Conclusion:**

Esketamine combined with ropivacaine hydrochloride used in labor analgesia can significantly reduce the incidence of postpartum depression after delivering without increasing related side effects, which may be related to the regulation of leptin, norepinephrine, and epinephrine in the serum.

**Trial registration:**

The trial was registered at the Chinese Clinical Trial Registry on 30/05/2022 (CTRI registration number—ChiCTR2200060387). URL of registry: https://www.chictr.org.cn/bin/home.

## Background

Maternal health has always been the focus. With the physical and psychological changes, the incidence of maternal postpartum depression(PPD) is about 3.5%~33% [1]. The causes of PPD are complex and related to maternal genetic, physiological, psychological, family, social and other factors. Studies have shown that maternal depressive mood, poor sleep quality, disharmonious family relations, lack of social support, trauma during pregnancy, and combined diseases are the major risk factors for PPD [2, 3]. PPD may occur from 1 to 6 weeks after delivering in both vaginal birth and cesarean delivery in both primiparous and multiparous women [4]. The maternal mental state not only affects their own health, but also is very important to the infants and the whole family. Events of suicide and infanticide often have been reported due to PPD [5].

At present, PPD is mainly treated by medication combined with psychological counseling. However, long-term medication may have adverse effects for lactating women on the infant’s neurological, motional and behavioral development [5, 6]. Therefore, prevention of PPD is even more important. Ketamine is an N-methyl-D-aspartate (NMDA) receptor antagonist, which is mostly used in anesthesia of pediatric, obstetric, and outpatient. In recent years, the antidepressant effect of ketamine has been paid more attention in the treatment of mental illness. Its application in patients with resistant depression has the advantage of rapidly improving depressive symptoms and significantly reducing the risk of suicide [7, 8]. Esketamine is an S-Enantiisomer of ketamine and has about twice the affinity to NMDA receptor (NMDAR), which is a hot topic of antidepressant research [9]. Our previous studies also suggested that esketamine prevent PPD in women underwent cesarean Sects. [10, 11]. However, the mechanisms of antidepressant effect of esketamine were unclear now. Additionally, with the widespread use of labor analgesia in obstetrics, it was also unknown whether esketamine was effective in such maternal postpartum depression. NMDAR are mainly distributed in the postsynaptic membrane of nerve cells in the central nervous system, so we speculate that esketamine for epidural labor analgesia can also exert antidepressant effects by binding to NMDA receptors on the spinal nerve roots [9]. Therefore, this study was designed to observe the effect of esketamine combined with ropivacaine hydrochloride on the incidence of maternal PPD after delivering and explore the related side effects and possible mechanisms to provide a reference for clinical prevention of PPD.

## Methods

### Ethics and participants

The Consolidated Standards of Reporting Trials (CONSORT) recommendations were followed in this study [12]. Ethical approval for this study (2021-03-031) was provided by the Institutional Ethics Committee of the Affiliated Jiangning Hospital of Nanjing Medical University. All women involved were informed of the proposal and gave their written, informed consent.

A total of 120 primiparous women who underwent labor analgesia from October 1st 2022 to March 31st 2023 in the Affiliated Jiangning Hospital of Nanjing Medical University were enrolled, with single term pregnancy, ASA physical status II, aged 24 to 36 years old, BMI 24 to 30 kg/m^2^.The exclusion criteria were as follows: (1) having mental disorder; (2) organic or pharmacogenic depression before delivering; (3) not suitable for transvaginal delivery; (4) combined pregnancy complications such as hypertension and diabetes; (5) combined with functional insufficiency of important organs such as heart, liver, kidney and others. (6) combined with coagulation disorders; (7) failure of epidural puncture; (8) changing to cesarean section.

### Sample size

**Fig. 1:**
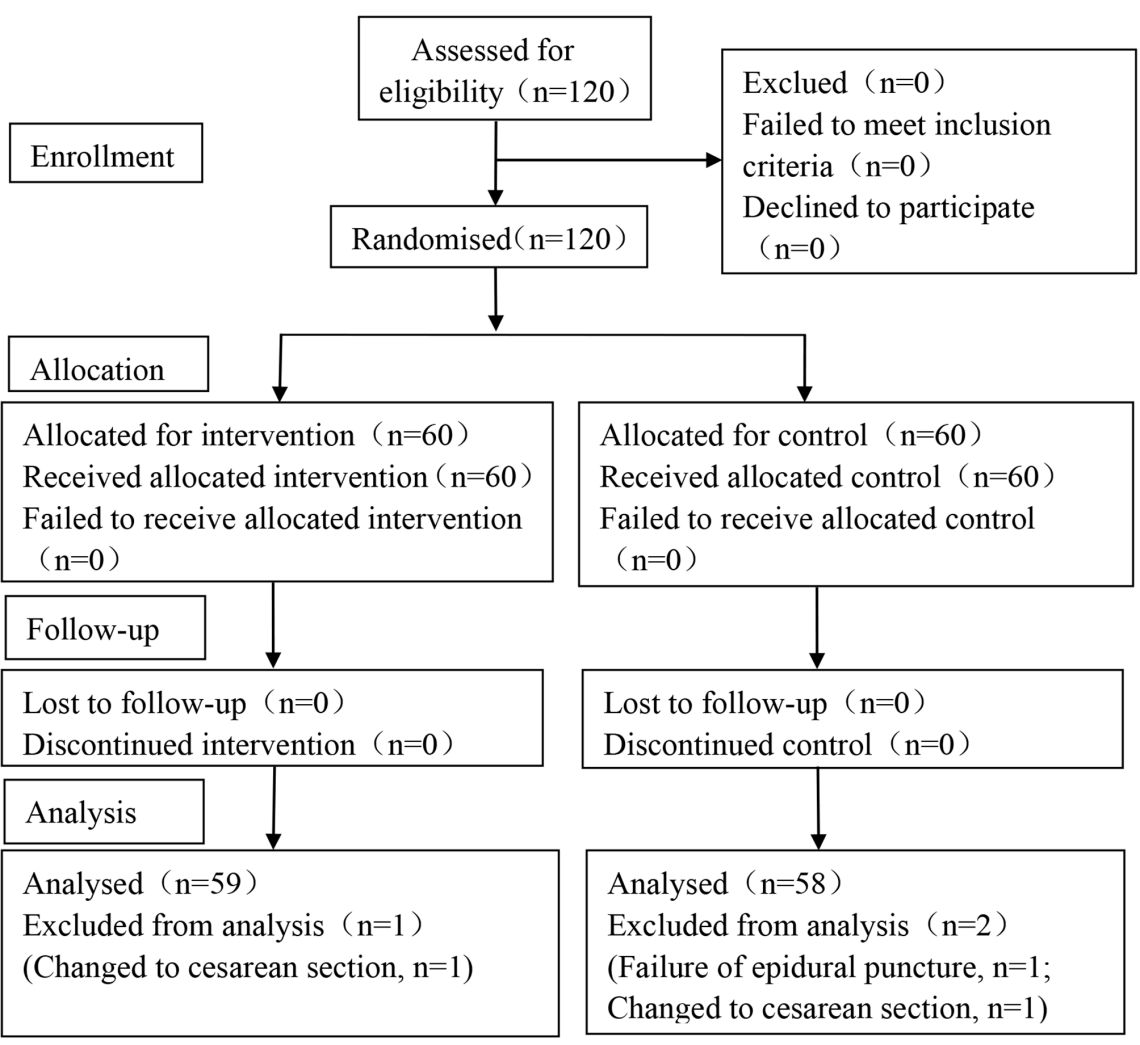
CONSORT flow diagram

Based on previous research [10, 11] and the results of our pre-experiment(10 participants in each group), the incidence of PPD at 6 weeks after delivering can be reduced by 10% in the esketamine group. A reduction rate of 10% was assumed with *α* = 0.05 and 1-β = 0.9, a sample size of at least 52 per group were needed according to the PASS sample size analysis software, version 2023 (NCSS, LLC, Connecticut, USA). Considering a 10% dropout rate, sixty samples for each group were designed in this study. Figure 1 showed the CONSORT flow diagram of the study participant’s recruitment.

### Randomization and allocation concealment

Patients were randomly assigned to one of two groups. Random tables were generated using SPSS 20.0. One hundred and twenty sealed envelopes were prepared by a statistician who does not participate in the study. The study was performed with neither patients nor the observers’ awareness of the group to which each patient belonged. To assure concealment of allocation, numbers were kept in sealed and opaque envelopes, which were opened by an anaesthesiologist who was not involved in this study.

### Interventions

After entering the delivery room, peripheral venous access was opened in all participants, maternal electrocardiography (ECG), heart rate (HR), noninvasive blood pressure measurement (NIBP), and pulse oximetry (SpO_2_) were monitored, and oxygen for 3 L/min by transnasal catheter were received. Epidural anaesthesia were operated in all women between L_2_ and L_3_ after cervical dilation up to 2 ~ 3 cm. After successful puncture, the epidural catheter was imbedded for 3.5 cm toward the head and 1% lidocaine was injected for 3 ml. The epidural analgesia pump was connected. Esketamine (0.2 mg/kg) combined with 0.75% ropivacaine hydrochloride (20 ml) were diluted by normal saline up to 100 ml in Group E, when only the equal dose of ropivacaine hydrochloride was used in Group C. All pumps were set with a bolus of 10 ml, continuous infusion amount of 8 ml/h, single dose of 4 ml, locking time of 15 min and stopped after fetal disengagement. All anesthesia-related operation were completed by the same anesthesiologist, and the delivering was supervised by the same group of obstetrician. Neither observers nor subjects were aware of the grouping condition, and the pump was configured by an anesthesia nurse who was not aware of the grouping condition as well.

### Main outcome measures

The occurrence of PPD at 1 week and 6 weeks after delivering were evaluated and recorded as the primary indicators in this study. The diagnostic criteria for PPD are usually defined in two steps: firstly, Edinburgh Postpartum Depression Scale (EPDS) was used to screen for suspicious patients (score > 9) [13]; secondly, suspicious patients were strictly tested according to the clinical protocol interview (SCID), the standard for the diagnosis of PPD was consulted by “the Diagnostic and Statistical Manual of Mental Disorders” (DSM-IV) [14]. The screening and diagnosis were determined by different physicians or above who did not know the grouping. The secondary indicators were as follows. The VAS score before analgesia (T_1_), 5 (T_2_), 10 (T_3_) and 20 (T_4_) minutes after analgesia were measured. The duration of the first and second stage of labor, the Apgar score of fetus at delivery, postpartum hemorrhage, consumption of esketamine and ropivacaine were recorded. All women were tested levels of leptin, norepinephrine (NE), and epinephrine (E) in peripheral venous blood before labor analgesia (when the woman was quiet in the ward), at 24 h, 1 week, and 6 weeks after delivering. The occurrence of side effects such as nausea, vomiting, dizziness, and nightmares for 48 h after delivering was recorded by anesthesia nurses who were not aware of the grouping as well.

### Statistical analysis

Data analysis was performed by the SPSS 20.0 statistical software package, version 20.0 (SPSS Inc., Chicago, IL, USA). Continuous variables were presented as mean ± SD or median with quartiles. Normally distributed data were analyzed by single factor variance analysis (one-way ANOVA). Non-normally distributed data were analyzed using the Mann-Whitney U test. The incidence of PPD and side effects were considered as categorical variables, which were presented as n(%) and analyzed with a χ^2^-test or Fisher’s exact test. It was considered statistically signifificant since a p-value < 0.05.

## Results

### Patient recruitment

In this study, 120 cases were initially screened, and 3 cases were excluded (In Group C, one case was excluded for failure of epidural puncture, and another participant was changed to cesarean section; in Group E, one case was excluded for changing to cesarean section). In total, 58 cases in Group C and 59 cases in Group E were included in the statistical analysis (Fig. 1) .

### Demographic data and patient characteristics

Women in two groups shared similar demographic characteristics (age, gestational week, BMI, duration of the first and second stage of labor, the Apgar score of fetus at delivery, postpartum hemorrhage and consumption of ropivacaine) (Table 1).

**Table 1:** Comparison of baseline characteristics in the two groups

Characteristic	Group E (n = 59)	Group C (n = 58)	*P-*value
Age (y)	27.6 ± 4.3	28.1 ± 3.9	0.556
Gestational age (weeks)	38.5 ± 2.6	38.8 ± 3.3	0.238
BMI (kg/m^2^)	28.2 ± 4.7	27.9 ± 5.1	0.318
Duration of the first stage of labor (min)	588.6± 93.2	603.1± 101.4	0.676
Duration of the second stage of labor (min)	108.8 ± 14.5	105.9 ± 12.7	0.412
Apgar score of fetus at delivery (points)	9.1 ± 1.2	9.0 ± 1.3	0.822
Postpartum hemorrhage (ml)	245.3 ± 37.6	233.6 ± 35.2	0.638
Consumption of esketamine (mg)	4.3± 1.1	0	< 0.001
Consumption of ropivacaine (mg)	42.5± 5.3	44.7± 6.1	0.121

Data are presented as mean ± SD. No significant differences between the two groups(*P* ≥ 0.05) except the consumption of esketamine (*P* < 0.001); mutual comparison were analyzed using single factor variance analysis(one-way ANOVA); BMI, Body mass index

### VAS score after labor analgesia

There were no significant difference of VAS score at T_1_ between the two groups. Compared with Group C, the VAS score at T_2_, T_3_ and T_4_ were significantly lower in Group E (*P* < 0.01) (Table 2) .

**Table 2:** VAS score after labor analgesia in the two groups

Group	T_1_	T_2_	T_3_	T_4_
Group E (n = 59)	8.6 ± 2.2	4.2 ± 2.3^a^	2.8 ± 1.8^a^	1.6 ± 1.1^a^
Group C (n = 58)	8.9 ± 1.9	5.5 ± 2.6	3.9 ± 2.0	2.9 ± 1.2
*P-*value	0.858	0.002	0.004	0.001

Data are presented as mean ± SD. Mutual comparison were analyzed using single factor variance analysis (one-way ANOVA); compared with group C, ^*a*^*P*<0.01. T_1_, before analgesia; T_2_, 5 min after analgesia; T_3_, 10 min after analgesia; T_4_, 20 min after analgesia; VAS, Visual analogue scale

### Incidence of PPD and side effects

Compared with Group C, the incidence of PPD in Group E was significantly lower after delivering (1 week: 3.4% vs. 17.2%, *P* < 0.01; 6 weeks: 5.1% vs. 20.7%, *P* < 0.01). There were no significant difference between the two groups on side effects, including, nausea, vomiting, dizziness, and nightmares for 48 h after delivering (Table 3) .

**Table 3:** Occurrence of PPD and side effects in the two groups

Group	PPD		nausea, vomiting	dizziness	nightmares
	1 week	6 weeks			
Group E (n = 59)	2(3.4)^a^	3(5.1)^a^	2(3.4)	2(3.4)	2(3.4)
Group C (n = 58)	10(17.2)	12(20.7)	3(5.2)	3(5.2)	1(1.7)
*P-*value	0.002	0.003	0.896	0.788	0.415

Data presented as n(%). Mutual comparison were analyzed using χ^2^-test; compared with group C, ^*a*^*P*<0.01. PPD, Postpartum depression

### Levels of leptin, NE and E

There were no significant difference of the levels of leptin, NE and E between the two groups before labor analgesia and 6 weeks after delivering. Compared with Group C, the levels of leptin were significantly higher at 24 h and 1 week after delivering in Group E (*P* < 0.01), while NE and E (*P* < 0.01) were lower at the same time (*P* < 0.01) (Table 4) .

**Table 4:** Levels of leptin, NE and E in the two groups

Time point	leptin (µg/L)			NE (nmol/L)			E (pmol/L)		
	Group E (n = 59)	Group C (n = 58)	*P-* value	Group E (n = 59)	Group C (n = 58)	*P-* value	Group E (n = 59)	Group C (n = 58)	*P-* value
Before analgesia	1.88 ± 0.46	1.92 ± 0.55	0.318	4.38 ± 0.76	4.55 ± 0.82	0.668	524 ± 36	531 ± 42	0.858
24 h	3.26 ± 0.62^a^	1.79 ± 0.32	0.002	3.13 ± 0.55^a^	4.20 ± 0.68	0.007	368 ± 27^a^	481 ± 36	0.003
1st week	3.50 ± 0.68^a^	1.71 ± 0.58	0.001	2.96 ± 0.49^a^	4.13 ± 0.70	0.005	336 ± 22^a^	475 ± 31	0.005
6th week	1.96 ± 0.51	1.83 ± 0.49	0.256	3.08 ± 0.52	3.32 ± 0.64	0.226	326 ± 25	340 ± 29	0.359

Data are presented as mean ± SD. Mutual comparison were analyzed using single factor variance analysis (one-way ANOVA); compared with group C, ^*a*^*P*<0.01. NE, Norepinephrine; E, Epinephrine

## Discussion

The “Diagnostic and Statistical Manual of Mental Disorders” (DSM-IV) commended by the American Psychiatric Association and the World Health Organization (WHO) defined perinatal depression as: no previous history of mental illness, during pregnancy or childbirth within 1 to 2 weeks, 4 to 6 weeks of depressive episode symptoms, such as emotional instability, severe anxiety, panic, crying and others [14].

The symptoms of PPD were more prominent in women who experience frequent pain during childbirth [15]. Although labor analgesia relieved maternal pain, psychological degeneration and emotional vulnerability after delivery also led to a high incidence of PPD in women underwent labor analgesia [16]. Therefore, the incidence of PPD at 1 week and 6 weeks after labor analgesia was observed in this study.

In recent years, ketamine had been found for its fast onset, long action time, and effect on refractory depression [7, 8]. Unlike the mechanism of 5-HT reuptake inhibitors, ketamine was an NMDAR antagonist, which provided a new target for the development of novel antidepressants. Berman et al. [17] found that depressive symptoms were significantly improved within 72 h after receiving a subanesthetic dose (0.5 mg/kg) of ketamine. Esketamine was an S-enantioisomer of ketamine, with approximately twice higher affinity to the NMDA receptor [9]. Tang et al. [18] reported that low-dose of esketamine (0.25 mg/kg) combined with opioids used for epidural analgesia after cesarean section achieved good analgesic effect without increasing side effects. Results of Gujral et al. [19] showed 0.2% ropivacaine with 0.5 mg/ml ketamine for epidural analgesia for postoperative epidural analgesia in adult patients undergoing elective lower limb surgery were also effective. Due to the advantages of motor-sensory block separation and less neurotoxicity, ropivacaine was widely used in the epidural labor analgesia [20], which was used alone or combined with opioids can meet the need of labor analgesia [21]. However, there were few reports on the use of esketamine combined with ropivacaine for epidural labor analgesia. In the study of Lou et al. [22], esketamine at a dose of 0.25−1.0 mg/ml combined with 0.075% ropivacaine diluted with normal saline to a total volume of 200 ml for labor analgesia achieved good analgesic effect. However, as the dose of esketamine increased, the rate of side effects such as dizziness were significantly increased. In contrast, the total volume of the epidural analgesia pump in our study was 100 ml, the background continuous infusion dose was set, and our main observation index was the incidence of PPD. Combined the above related studies, a small dose of esketamine at 0.2 mg/kg was designed in this study based on considerations of patients’ safety, which also showed no significant side effects. Bahji et al. [23] reported that esketamine rapidly improved depressive symptoms from 2 to 4 h after intravenous injection. Esketamine had been approved to the treatment of depression by FDA in the US. The study of Swainson et al. [24] showed that intravenous injection of esketamine was similar with ketamine for antidepressant effects, but the former might be superior of the long-term safety data. Our previous studies suggested that combined use of esketamine in maternal postoperative intravenous analgesia for cesarean section can reduce the occurrence of PPD, and prophylactic intravenous esketamine after delivery of the fetus also lead to a lower incidence of PPD in women underwent cesarean Sects. [10, 11]. The results of this study showed that the incidence of PPD was significantly lower in Group E, which confirmed the antidepressant effect of esketamine used in labor analgesia. The antidepressant effect of esketamine could last from 3 to 7 days, which might be related to the antidepressant effect of its metabolite (normethylketamine) [25]. In addition, studies have shown that the use of opioids combined with ropivacaine hydrochloride in labor analgesia provided better analgesia, but there may be some risks such as affecting the breathing of the fetus [26, 27]. In the present study, esketamine also played a synergistic analgesic role in labor analgesia, and having no effect on the Apgar score of fetal at delivering. Because of the different mechanisms of analgesic action of esketamine and opioids, we did not compare their synergistic analgesic effects in this study.

In this study, three objective indicators of leptin, NE and E were introduced to analyze their relationship with PPD. As endocrine hormones, NE and E can affect the the bodys’ mood by modulating the sensitivity of neuronal synapses and neurotransmitters [28]. NE can return to the synaptic space under the action of norepinephrine transporter protein, and also form E by acting on phenylmethylamine-N-methyltransferase, which easily lead to tension and fear [29]. It had been reported that due to the changes in stress and hormone levels during pregnancy, the blue spot located in the brain stem was activated, thus releasing a large number of NE to participate in the regulation of the nervous system, which would be easy to cause tension and depression [30]. Leptin is a kind of hormones produced and secreted by adipose tissue that can bind to leptin receptors across the blood-brain barrier. Leptin receptors are mainly located in brain regions related to emotional control, such as the hippocampus, hypothalamus, and cerebral cortex, and they are partly found in serotonergic neurons in the brainstem [31]. The binding of leptin to the leptin receptor lead to changes in the structure and function of the hippocampus and the cerebral cortex to regulate the bodys’ mood [32]. The results of this study showed that esketamine had a short onset time. Levels of leptin decreased ,while NE and E were increased at 24 h after delivering, which lasted 1 week. The antidepressant mechanism may be the target of esketamine for monoaminergic nervous system, can affect the brain stem blue spot, and further affect the noradrenergic system, promote the transcription and expression of the gene, increase the synthesis of transporter, resulting in NE migration from plasma membrane to cytoplasm, decrease the concentration of serum NE and E [33]. Studies have shown that esketamine can also act with leptin receptors through the glutamate system to relieve tension and depression [34]. In addition, the antidepressant effects of esketamine may also be related to the following. The radioligand-binding properties of NMDAR are specifically altered in depressed patients. Esketamine can sustainability block NMDAR isoforms and the elongation factor 2 (eEF2) kinase in eukaryotic cells, dephosphorylate eEF2, increase the expression of tropommyosin receptor kinase B (TrkB), and ultimately increase the release of neurotrophic factor (BDNF) to improve neural plasticity and synapse formation [35]. Esketamine can also reduce the inhibition of the presynaptic glutamatergic pathway by inhibiting the activity of γ-aminobutyric acid (GABA), leading to an increased release of presynaptic membrane glutamate, resulting in an excitation of a-amino−3-hydroxy−5-methyl−4 isooxazole propionic acid receptor (AMPAR), following a series of intracellular biochemical reactions to produce antidepressant effects [36]. In addition, a recent animal experiment showed that esketamine can inhibit autophagy through mTOR-BDNF signaling, thus reducing neuroinflammation induced by lipopolysaccharide (LPS), mainly decreasing inflammation-related cytokines (TNF-, IL−1, IL−6), apoptotic factors and autophagic marker levels, to deliver antidepressant effects [37].

As an anesthetic drug, esketamine was used many years, but there were no sufficient data for the prevention or treatment of psychiatric diseases. Considering the possible risk of hallucinogenic effects and abuse of esketamine, the dose used in this study was slightly lower than the subanesthetic dose, and labor analgesia was stopped after fetal delivery. Results of follow-up showed that there were no significant difference of the occurrence of side effects for 48 h after labor analgesia between the two groups.

There were some limitations in this study, such as the strong subjectivity of the PPD evaluation by EPDS. To minimize the subjective differences, the PPD valuator in this study was the same physician who did not participate in the specific study. Furthermore, the objective indicators such as levels of leptin, NE, and E were also combined in this study, which could better reflect maternal depression. In addition, the severity of PPD was not observed in the study, which would be the content of further studies.

## Conclusion

Esketamine combined with ropivacaine hydrochloride used in labor analgesia can significantly reduce the incidence of postpartum depression at 1 week and 6 weeks after delivering, without increasing related side effects. The regulation of leptin, norepinephrine, and epinephrine in the serum may be related to esketamine’s antidepressant effect.

In this study, 120 cases were initially enrolled, and 3 cases were excluded. In total, data of 58 cases in Group C and 59 cases in Group E were analyzed. CONSORT, the Consolidated Standards of Reporting Trials.

## Data Availability

The datasets used and analyzed during the current study are available from. the corresponding author on reasonable request. Qi Wang, e-mail: 848892149@qq.com.

## Abbreviations

PPD: Postpartum depression
ASA: American society of Aneshesiologists physical status
VAS: Visual analogue scale
NMDA: N-methyl-D-aspartate
NMDAR: N-methyl-D-aspartate receptor
CONSORT: The Consolidated Standards of Reporting Trials
EPDS: The Edinburgh Postpartum Depression Scale
NE: Norepinephrine
E: Epinephrine
BMI: Body mass index
